# Mental incapacity in high office: historical precedents and unresolved challenges for modern governance

**DOI:** 10.1192/bjb.2025.10169

**Published:** 2025-12

**Authors:** Alexander Smith, Dinesh Bhugra, Juan Graña, Agustín Artese, Michael Liebrenz

**Affiliations:** Department of Forensic Psychiatry, University of Bern, Bern, Switzerland; Institute of Psychiatry, Psychology and Neuroscience, King’s College London, London, UK; Institute of Latin American and Caribbean Studies, University of Buenos Aires, Buenos Aires, Argentina

In 2024, a faltering debate performance effectively ended the re-election prospects of the then-president of the United States, Joe Biden, intensifying scrutiny of his ‘fitness to govern’.^1,2^ Subsequent reports claimed that Mr Biden’s aides had concealed signs of his apparent cognitive decline, prompting a post-presidency inquiry into his mental health and decision-making.^2^ Meanwhile, the incumbent American president Donald Trump and other modern world leaders have attracted similarly controversial psychopathological speculation.^1,2^ Together, such discourse has reanimated concerns about psychiatric vulnerabilities among heads of government, which have long resonated throughout disparate eras and political systems.^1,3–5^

Centuries earlier, King Christian VII (1749–1808) nominally ruled Denmark–Norway, despite exhibiting psychiatric symptoms. As the monarch’s impairments worsened, the royal physician, Johann Friedrich Struensee (1737–1772), became a trusted advisor and then the *de facto* regent.^3^ Ultimately, Struensee’s reformist agenda and his affair with Christian’s queen, Caroline Matilda (1751–1775), would result in his execution.^3^ The reign of Christian’s near-contemporary King George III of Great Britain (1738–1820) was also affected by mental ill-health, eventually necessitating a regency.^4^

Later, comparable cases transpired across 20th-century democracies. For example, British prime minister Winston Churchill (1874–1965) allegedly exhibited depressive symptoms (the so-called ‘black dog’) and harmful alcohol use.^4^ Separately, Ronald Reagan (1911–2004) and Urho Kekkonen (1900–1986) are believed to have had dementia when serving as presidents of the United States and Finland, respectively.^1,4^ However, psychiatric insights regarding these and other political actors have typically only emerged retrospectively or unofficially, or remain uncorroborated.^1,4^

While recent officeholders have commendably begun to discuss their occupational stressors, sensitivities and stigma endure, as reinforced by the paucity of data on the mental well-being of politicians internationally.^1,4^ Although psychiatric disorders in themselves should not (and do not) inherently negate effective leadership, the risks of serious functional impairments in high office, particularly when they are not transparently addressed, invoke pertinent questions about societal safeguards. Still, in many democracies, legal provisions surrounding mental incapacity are poorly defined, untested or altogether absent, as are standards for measuring ‘fitness to govern’.^1,4^

These complexities are likely to be amplified in autocratic and authoritarian regimes, which have proliferated in recent years and routinely eschew accountability and suppress open expression. Throughout these settings, statutory measures could be more likely to be subverted (assuming they exist at all) and medical conditions ‘covered up’. This was exemplified during the later presidency of Saparmurat Niyazov (1940–2006), whose cult of personality and centralised authority precluded any meaningful domestic pressure in Turkmenistan about his increasingly erratic actions and public absences.^5^

Globally, preventive proposals abound to mitigate these challenges, including compulsory and impartial psychiatric examinations of leaders, mandatory health disclosures and constitutional mechanisms.^1^ The practicality (and wisdom) of these initiatives is debatable, and sociopolitical and legal conventions would determine their applicability. Nevertheless, past precedents have demonstrated the potentially destabilising effects of mental incapacity at the apex of power, which should warrant serious reflection.^4^ In today’s era of ‘strongmen’, democratic regression and nuclear brinkmanship, the psychiatric community, policy makers and wider societies must be attentive to these possibilities; history, after all, does not repeat itself, but it quite often rhymes.
